# LncPrep + 96kb 2.2 kb Inhibits Estradiol Secretion From Granulosa Cells by Inducing EDF1 Translocation

**DOI:** 10.3389/fcell.2020.00481

**Published:** 2020-06-30

**Authors:** Fen Feng, Jing Wang, Riqiang Bao, Long Li, Xiating Tong, Suo Han, Hongdan Zhang, Weihui Wen, Li Xiao, Chunping Zhang

**Affiliations:** ^1^Department of Cell Biology, College of Medicine, Nanchang University, Nanchang, China; ^2^Department of Microbiology, College of Medicine, Nanchang University, Nanchang, China; ^3^Joint Program of Nanchang University and Queen Mary University of London, College of Medicine, Nanchang University, Nanchang, China

**Keywords:** estradiol, lncPrep + 96kb, EDF1, aromatase, granulosa cell

## Abstract

LncPrep + 96kb is a novel long non-coding RNA expressed in murine granulosa cells with two transcripts that are 2.2 and 2.8 kb in length. However, the potential roles of lncPrep + 96kb in granulosa cells remain poorly understood. In this study, we investigated the effect of the lncPrep + 96kb 2.2 kb transcript on granulosa cells through the overexpression and knockdown of lncPrep + 96kb 2.2 kb. We found that lncPrep + 96kb 2.2 kb inhibited aromatase expression and estradiol production. Endothelial differentiation-related factor 1 (EDF1) is an evolutionarily conserved transcriptional coactivator. We found that EDF1 knockdown inhibited aromatase expression and estradiol production. The RNA immunoprecipitation results also showed that lncPrep + 96kb 2.2 kb can bind to EDF1 and that overexpression of lncPrep + 96kb 2.2 kb induced the translocation of EDF1 from the nucleus to the cytoplasm. The CatRAPID signature revealed that the 1,979–2,077 and 603–690 nucleotide positions in lncPrep + 96kb 2.2 kb were potential binding sites for EDF1. We found that mutating the 1,979–2,077 site rescued the effects of lncPrep + 96kb 2.2 kb on aromatase expression and estradiol production. In conclusion, we are the first to report that specific expression of lncPrep + 96kb 2.2 kb in granulosa cells inhibits the production of estradiol by influencing the localization of EDF1 in granulosa cells. The 1,979–2,077 site of lncPrep + 96kb 2.2 kb contributes to the ability to bind to EDF1.

## Introduction

Ovarian follicles are the basic functional units of ovaries. In mammals, ovarian follicular development is a continuous process throughout the reproductive life span. Follicles progress through the primordial, primary, and secondary stages before developing an antral cavity. With further growth and differentiation, preovulatory follicles form, and oocytes are released after being stimulated with luteinizing hormone (LH) (Zhang et al., 2011).

The growth and maturation of follicles are always accompanied by granulosa cell proliferation and differentiation (Johnson, 2015). Granulosa cells can acquire specific functional characteristics after differentiation, including the expression of specific receptors and the secretion of progesterone and estrogen to coordinate oocyte maturation and subsequent events such as ovulation, fertilization, and early embryo development (Sasson et al., 2004; Wang Q. et al., 2013).

Estrogens are the main steroids produced by granulosa cells in mature follicles. Ovarian estrogens play key roles in the female reproductive system and secondary sexual characteristics. In the reproductive system, estrogens regulate follicular development (Baba et al., 2017). It is well known that ovarian follicles can develop until the preantral and early antral stages independently of follicle-stimulating hormone (FSH). Estrogens stimulate the growth of primary and secondary follicles until the preantral stage (Laven and Fauser, 2006). Studies have shown that estrogens increase the number and size of ovarian follicles *in vivo* and the size of rat, mouse, and bovine follicles *in vitro* (Rosenfeld et al., 2001). In a steroid-depleted milieu, estradiol also improved secondary follicle survival, growth, antrum formation, and oocyte health in adult rhesus macaques (Ting et al., 2015). However, other studies showed that estrogens had inhibitory effect on follicle development in some species. *In vivo* diethylstilbestrol (DES) exposure induces atresia of the dominant follicles in rats and monkeys (Hutz et al., 1986). *In vitro* high-dose estradiol exposure delayed or inhibited oocyte meiotic maturation during murine secondary follicle culture (Tarumi et al., 2014). Therefore, the effects of estrogens can be stimulatory or inhibitory, depending on the species, dose, and duration of exposure. Studies have also shown that estrogens can lead to synchronization of follicular development in prepubertal mice (Wu et al., 2015). Studies of the underlying mechanism have shown that estrogens stimulate the proliferation of granulosa cells, have antiapoptotic effects on granulosa cells, and control follicular neovascularization (Li et al., 2013; Chou and Chen, 2018). Besides the effects on follicle development, estrogens exert negative feedback on LH production in the early stage of the menstrual cycle. When estrogen levels reach a critical level, they exert positive feedback on LH production and ovulation (Drummond, 2006; Holesh et al., 2020).

Estradiol, one main estrogen, is synthesized by granulosa cells. Aromatase in granulosa cells converts androgens (from theca cells) into estradiol. The loss of this enzyme is responsible for mouse infertility (Fisher et al., 1998). The expression of aromatase is controlled in a cell-specific, temporal, and spatial manner, which limits estradiol production. Studies have shown that aromatase is expressed at low levels in the granulosa cells of small growing follicles during the fetal and neonatal periods, and estradiol production is limited at this stage. In 21-day-old rats, aromatase is no longer expressed in growing follicles, but is expressed in only healthy large antral follicles and preovulatory follicles. Steroidogenesis can occur in only the postpubertal period. Therefore, the granulosa cells of large antral follicles and preovulatory follicles are the main cells that produce estradiol (Guigon et al., 2003; Sakurada et al., 2006; Stocco, 2008; Dewailly et al., 2016).

It is well accepted that FSH is the major inducer of aromatase activity in granulosa cells. In addition, other endocrine factors, paracrine factors, and autocrine factors all contribute to estrogen production (Stocco, 2008; Hobeika et al., 2019). For example, insulinlike growth factor 1 (IGF1) alone increased estradiol production to a level that was comparable to the level induced by FSH. Follicle-stimulating hormone and IGF1 have a synergistic effect on estradiol production (Sahmi et al., 2014). The proximal ends of the aromatase promoter contain a number of fairly well-conserved regulatory elements, including steroidogenic factor 1 (SF1, NR5A1), liver receptor homolog 1 (LRH-1, NR5A2), adenosine 3,5-cyclic monophosphoric acid (cAMP), and forkhead transcription factor 2 (Foxl2), which contribute to the regulation of aromatase and estradiol production (Stocco, 2008).

ENCODE (Encyclopedia of DNA Elements) shows that the vast majority (80.4%) of the human genome can be transcribed into RNA. Non-coding RNAs account for most of the genome (Consortium, 2012; Dhahbi, 2014) and include small non-coding RNAs and long non-coding RNAs (lncRNAs). MiRNAs have been demonstrated to play pivotal roles in regulating the sequential recruitment, selection, and development of follicles, atresia, and ovulation (Hossain et al., 2012; Imbar and Eisenberg, 2014; Li et al., 2015). Over the last few years, lncRNAs have been demonstrated to have important biological functions, which are involved in gene expression, and interact directly with biological macromolecules (DNAs, RNAs, and proteins) (Mercer and Mattick, 2013). Long non-coding RNAs have notably decreased sequence conservation compared to protein coding genes, but their secondary structure, rather than their sequence, usually dictates their function (Pang et al., 2006; Struhl, 2007; Mercer and Mattick, 2013). Long non-coding RNAs are implicated in a variety of biological processes, including dosage compensation, imprinting, cell cycle control, cell development, and gametogenesis (Yang et al., 2014). Moreover, the spatial and temporal-specific expression of lncRNAs shows a strong association with the cell differentiation process (Cesana et al., 2011; Wang Y. et al., 2013). Studies have shown that some lncRNAs are highly expressed in the ovaries. For instance, Neat1 plays an important role in corpus luteum formation, as Neat1 knockout mice fail to become pregnant (Nakagawa et al., 2014). Long non-coding RNA SRA was also demonstrated to promote growth, inhibit apoptosis, and induce the production of estradiol and progesterone in granulosa cells. Abnormal expression of lncRNA SRA may be a risk factor for polycystic ovary syndrome (Li et al., 2018). Long non-coding RNA-Amhr2 regulates Amhr2 gene activation in mouse ovarian granulosa cells (Kimura et al., 2017).

A novel lncRNA, termed lncPrep + 96kb, has been identified as the product of conserved non-coding sequences in the intergenic region adjacent to prolyl oligopeptidase (Matsubara et al., 2014). LncPrep + 96kb consists of two transcripts of different sizes, 2.2 and 2.8 kb. Matsubara et al. (2014) found that this lncRNA was highly expressed in murine granulosa cells. However, the physiological function of lncPrep + 96kb in granulosa cells is still unclear. In this study, we found that lncPrep + 96kb was mainly expressed in granulosa cells of small growing follicles and identified the inhibitory effect of lncPrep + 96kb 2.2 kb on aromatase expression and estradiol production in granulosa cells. We also further explored the possible underlying mechanism.

## Materials and Methods

### Animals

Kunming mice were purchased from the Animal Facility of Nanchang University and housed in a temperature- and light-controlled facility with free access to water and food. The experimental protocols were approved by the ethical committee of Nanchang University (SYXK2015-0001, December 29, 2015).

### Chemicals

An antibody against endothelial differentiation-related factor 1 (EDF1) (BS70093) was obtained from Bio World, Nanjing, China. An antiaromatase antibody (BA3704) was obtained from BOSTER, Wuhan, China. Real-time polymerase chain reaction (PCR) mix (208054) was obtained from QIAGEN, Germany. ANTI-FLAG^®^M2 magnetic beads (M8823) were obtained from Sigma, St. Louis, MO, United States. Phusion^®^ high-fidelity DNA polymerase and endonuclease were obtained from New England Biolab, Ipswich, MA, United States. FuGENE 6 transfection reagent (E2691) was purchased from Promega, Madison, WI, United States. An anti–β-actin antibody (HC201), anti-Flag antibody (HT201), and a CCK8 kit (FC101) were obtained from TransGen Biotech, Beijing, China. The digoxigenin (DIG) RNA labeling mix (11277073910) and anti-DIG-Fab antibody (110932749100) were obtained from Roche, Mannheim, Germany. pLV[shRNA]-EGFP:T2A:Puro-U6 > mEdf1 plasmid was obtained from Vector Builder, Guangzhou, China.

### *In situ* Hybridization

The probe sequence of lncPrep + 96kb was amplified using Phusion^®^ high-fidelity DNA polymerase and ligated into the pGEMT vector. The DIG-labeled RNA probe was synthesized as previously reported (Zhang et al., 2011). Ovaries were fixed in 4% paraformaldehyde for 12 h and embedded in paraffin. Then, 5 μm sections were cut. After deparaffinization and rehydration, the sections were permeabilized with proteinase K (10 mg/L) in proteinase K buffer for 25 min at 37°C, incubated in prehybridization buffer for 4 h at 42°C, and incubated with the hybridization solutions, which were composed of DIG-labeled antisense RNA probes and hybridization buffer, at 56°C for 18 h. Detection of the hybridized probe was performed using an alkaline phosphatase–conjugated anti-DIG antibody (Fab fragment). After washing, the color was developed with color-generating solution (4-nitroblue tetrazolium chloride and 5-bromo-4-chloro-3-indolyl-phosphate,4-toluidinesalt) for 2 h.

### CatRAPID Prediction of RNA–Protein Interactions

We used the CatRAPID online server to evaluate the interaction between lncPrep + 96kb 2.2 kb and EDF1 based on the secondary structure, hydrogen bonding, and van der Waals interactions, which rapidly predict the interactions between RNA and proteins (Agostini et al., 2013; Livi et al., 2016; Shang et al., 2019). The binding capacity of the EDF1 protein was estimated via algorithms using the CatRAPID signature module. The binding sites between lncPrep + 96kb 2.2 kb and EDF1 were evaluated by the CatRAPID fragments module.

### Plasmid Constructs

To construct the lncPrep + 96kb 2.2 kb overexpression plasmid, the whole sequence of lncPrep + 96kb 2.2 kb was amplified by PCR, digested with the *Kpn*I and *Eco*RI restriction enzymes, and cloned into the PCDNA3.0 plasmid vector. Bridge PCR was employed to construct the lncPrep + 96kb 2.2 kb mutation plasmids. The primers are shown in Table 1. The guide RNA (gRNA) targeted against lncPrep + 96kb was cloned into the pSpCas9(BB)-2A-GFP vector (PX458). We designed the gRNAs online using a web server^1^, and the oligonucleotide pairs for each gRNA were subsequently synthesized (Table 2). The PX458 plasmid was digested with *Bbs*I and annealed to the gRNAs. DNA sequencing was used to confirm successful insertion.

**TABLE 1:** Oligonucleotides used for constructing plasmids.

**Gene**	**Sense and antisense primer sequence**
lncPrep + 96kb 2.2 kb forward	5′-TTGGTACCAGCTTGTGTATTGCTCATAT-3′
lncPrep + 96kb 2.2 kb reverse	5′-GGAATTCTTTGCTTTTTAATTTTTATTTG-3′
lncPrep + 96kb 2.2 kb mutation (1,979–2,066) reverse	5′-GGAATTCAGACAACAGGAGCCCCTG-3′
Bridge 603 reverse primer	5′-AGCCTTTATCCTCTGCCATGTGTCCT CTATTCTGTACTC-3′
Bridge 690 forward primer	5′-AGAGGATAAAGGCTAAGAAC-3′
lncPrep + 96kb probe forward	5′-GGAGCAGCTGAGATAGAAGC-3′
lncPrep + 96kb probe reverse	5′-GGCCACCCATTTCTACTTAC-3′

**TABLE 2:** Sequences of gRNAs designed online.

**Name**	**Sequence**
lncPrep + 96kb gRNA forward	5′-ACACCGGTAATCCTAACGCCACGAAG-3′
lncPrep + 96kb gRNA reverse	5′-AAAACTTCGTGGCGTTAGGATTACCG-3′

### Granulosa Cell Culture and Transfection

The isolation of granulosa cells was performed as follows. Female mice (21 days) were injected with 5 IU pregnant mare serum gonadotropin (PMSG), and the ovaries were collected 48 h later. The fat and capsule tissues adhering to the ovaries were removed. Following two washes, the ovaries were mechanically dissected with a syringe needle in sterile phosphate-buffered saline (PBS). The suspension was collected and filtered through 75 μm strainers to remove debris and was centrifuged at 500 *g* for 5 min. The cells were resuspended in Dulbecco medium eagle medium/F12 culture medium supplemented with 5% fetal bovine serum, 100 IU/mL penicillin, and 100 μg/mL streptomycin sulfate. The cells were seeded and cultured overnight until they adhered to the cell culture plate. The plasmids were transfected into the granulosa cells using FuGENE 6 when the cell confluence reached 70%. After 8 h, the medium was changed. Then 100 nM androstenedione was added into the medium as a substrate of estradiol. The medium was harvested for estradiol examination, and the cells were lysed for mRNA and protein extraction at the indicated times.

### Cell Viability Assay

Granulosa cells were seeded in 96-well plates at 5 × 10^3^ cells per well. The cells were transfected with the indicated plasmids for 48 h. Ten microliters of CCK-8 reagent was added to each well and incubated for 2 h. The absorbance of reduced WST-8(2-(2-methoxy-4-nitrophenyl)-3-(4-nitrophenyl)-5-(2,4-disulfophenyl)-2H-tetrazolium) was measured at 450 nm using an enzyme-linked immunosorbent assay plate reader.

### Radioimmunoassay

The cultured granulosa cells were transfected with different plasmids. The supernatant was harvested at different times and stored at −80°C for subsequent hormone measurement. Estradiol was assessed by a commercial laboratory (Beijing Sino-uk Institute of Biological Technology, Beijing, China) using a commercial radioimmunoassay kit. For the kit, the cross-reactivities of steroid hormones and other peptides are less than 4%. The intra-assay and inter-assay variation coefficients did not exceed 10%. The sensitivity of estradiol detection was 2 pg/mL.

### Immunofluorescence

Granulosa cells were fixed in 4% paraformaldehyde for 30 min. After washing with PBS, the slides were incubated with 5% bovine serum albumin in PBS for 30 min at room temperature to reduce non-specific binding and then incubated with a primary antibody against EDF1 (2.5 μg/mL) at 4°C overnight. After three rinses in PBS, the slides were incubated for 1 h at room temperature with goat anti–rabbit immunoglobulin G conjugated to fluorescein isothiocyanate. DAPI (4′,6-diamidino-2-phenylindole) was used to stain the nuclei. For the negative control, the slides were incubated with PBS instead of the primary antibody. Other procedures were exactly the same.

### RNA Extraction and Real-Time PCR

Total RNA was isolated with TRIzol reagent according to the manufacturer’s instructions. Two micrograms of total RNA was used to synthesize cDNA using reverse transcriptase. Real-time PCR was carried out in a 20 μL reaction volume that consisted of 10 μL 2X Brilliant SYBR Green qPCR Master Mix, 1 μL cDNA, 0.5 μL primers, and 300 nM ROX reference dye using an ABI thermal cycler 7500 under the following conditions: 94°C for 30 s and 40 cycles of 94°C for 5 s and 60°C for 60 s. Melting curve analysis was conducted to analyze the purity of the products after real-time PCR. Experiments were repeated three times, and each gene was analyzed in parallel. The sequences for the specific primers are listed in Table 3. The threshold cycle (Ct) values were normalized to the mRNA expression level of glyceraldehyde-3-phosphate dehydrogenase (GAPDH) and calculated with the ΔΔCt method, which was then expressed as 2^–ΔΔ*Ct*^ for the sake of analyzing the fold change.

**TABLE 3:** Oligonucleotides used for real-time PCR.

**Gene**	**Sense and antisense primer sequence**
GAPDH forward	5′-TCCTTGGAGGCCATGTAGGCCAT-3′
GAPDH reverse	5′-TGATGACATCAAGAAGGTGGTGAAG-3′
Aromatase forward	5′-GCACAGTCACTACATCTCCCGA-3′
Aromatase reverse	5′-CACACAAACTTCCACCATTCGA-3′
EDF1 forward	5′-CTGCATCACGACAGGGTGAC-3′
EDF1 reverse	5′-ATAGTCTGCGATGACTTGCGG-3′

### Protein Extraction and Western Blotting

Granulosa cells were washed with PBS and lysed in RIPA (radioimmune precipitation assay) lysis buffer containing complete Mini Protease Inhibitor Cocktail tablets (Roche, Mannheim, Germany). A Bradford assay (Bio-Rad Laboratories, Hercules, CA, United States) was employed to estimate the protein concentration. The proteins were separated on 12% sodium dodecyl sulfate–polyacrylamide gel electrophoresis gels and transferred to nitrocellulose membranes using a 200 mA constant current for 2 h. The blots were blocked with 5% skim milk for 1 h and incubated overnight at 4°C with a primary antibody (500 ng/mL for aromatase and EDF1). The membranes were washed in Tris-buffered saline Tween-20 three times and incubated with a horseradish peroxidase–conjugated secondary antibody at room temperature for 1 h. ECL substrate was used to visualize the target bands, and the band intensity was quantified by densitometry (Bio-Rad Image Lab). β-Actin served as an internal control.

### RNA Immunoprecipitation and Mass Spectrometry

The interaction between lncPrep + 96kb 2.2 kb and protein was detected by RNA immunoprecipitation (RIP) based on the specific interaction between the MS2 coat protein and MS2 binding sites (MS2bs) as reported (Li et al., 2017). The pcDNA3.0-12 × MS2bs and pcDNA3.0-Flag-2 × MS2 plasmids were gifts from Professor Zheng Xiaofei (Beijing Institute of Radiation Medicine, Beijing, China). We amplified the full length of lncPrep + 96kb 2.2 kb and cloned it into pcDNA3.0-12 × MS2bs, which was named pcDNA3.0-lncPrep + 96kb 2.2 kb-12 × MS2bs. The pcDNA3.0-Flag-2 × MS2 and pcDNA3.0-lncPrep + 96kb 2.2 kb-12 × MS2bs plasmids were cotransfected into granulosa cells. After 48 h, the cells were washed with PBS and then cross-linked with UV light for 15 min. Then the cells were lysed in Pierce IP Lysis buffer containing protease, RNA inhibitors, and DNase I for 30 min on ice, and centrifuged at 12,000 *g* for 10 min. ANTI-FLAG^®^ M2 magnetic beads were washed with TBS solution, and the cell lysates were added and incubated overnight at 4°C. The FLAG-tagged proteins were immunoprecipitated from the supernatants with ANTI-FLAG^®^ M2 magnetic beads. After three washes with a low-salt wash buffer (50 mM Tris-Cl, pH 7.4, and 150 mM NaCl), the magnetic beads were boiled in loading buffer, and the protein samples were further analyzed through mass spectrometry (Changya Biotech Co., Ltd., Shanghai, China). Western blot analysis was further used to confirm the results of mass spectrometry.

### Statistical Analysis

All statistical analyses were performed using GraphPad Prism v6.01 (GraphPad Software Inc., San Diego, CA, United States). Data were presented as the mean and standard error of the mean. An independent-samples *t*-test was used for statistical comparisons between two groups. One-way analysis of variance followed by the Student–Newman–Keuls test was used for statistical comparisons among multiple groups. A *p*-value less than 0.05 was deemed statistically significant.

## Results

### Lncprep + 96kb Is Spatiotemporally Expressed in Murine Ovaries

**FIGURE 1:**
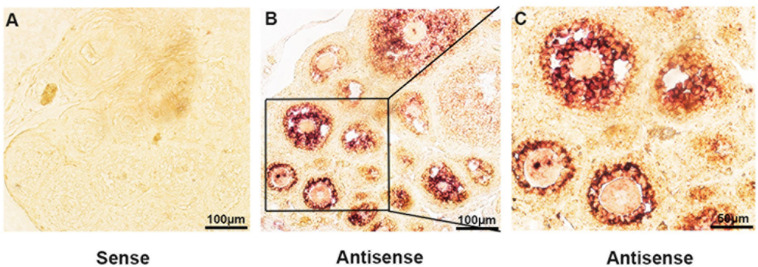
The expression of lncPrep + 96kb in murine ovaries. The negative control is shown in **(A)**. **(B,C)** Show the positive signal of lncPrep + 96kb. Scale bar = 100 μm.

The expression of lncPrep + 96kb in ovaries was detected through *in situ* hybridization. We found that the lncPrep + 96kb signal was mainly located in the granulosa cells of primary and secondary follicles. A weak signal was also detected in the granulosa cells of antral follicles (Figure 1).

### LncPrep + 96kb 2.2 kb Decreases the Production of Estradiol and Inhibits the Expression of Aromatase

**FIGURE 2:**
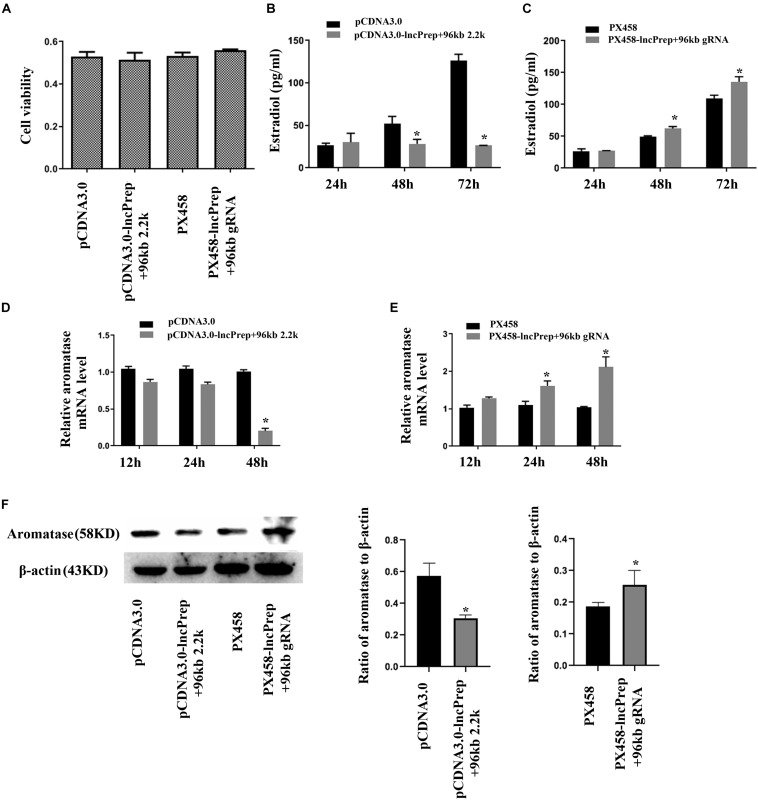
Estradiol production and aromatase expression are regulated by lncPrep + 96kb 2.2 kb. **(A)** Shows the effect of lncPrep + 96kb 2.2 kb on cell proliferation. **(B,C)** Show estradiol production after the overexpression and knockdown of lncPrep + 96kb 2.2 kb. **(D,E)** Show the mRNA expression of aromatase after overexpression and knockdown of lncPrep + 96kb 2.2 kb. **(F)** Shows the protein expression of aromatase after overexpression and knockdown of lncPrep + 96kb 2.2 kb. The experiment was independently repeated for three times. *p < 0.05, which was a specific two-group comparison.

To observe the function of lncPrep + 96kb 2.2 kb in granulosa cells, we overexpressed and knocked down the expression of lncPrep + 96kb 2.2 kb. The CCK-8 assay results showed that the proliferation of granulosa cells was not affected (Figure 2A). Overexpression of lncPrep + 96kb 2.2 kb inhibited the production of estradiol, and knockdown of lncPrep + 96kb 2.2 kb promoted the secretion of estradiol (Figures 2B,C). The expression of aromatase was negatively regulated by lncPrep + 96kb 2.2 kb at the mRNA and protein levels (Figures 2D–F).

### Lncprep + 96kb 2.2 kb Binds to EDF1 and Induces the Translocation of EDF1

**FIGURE 3:**
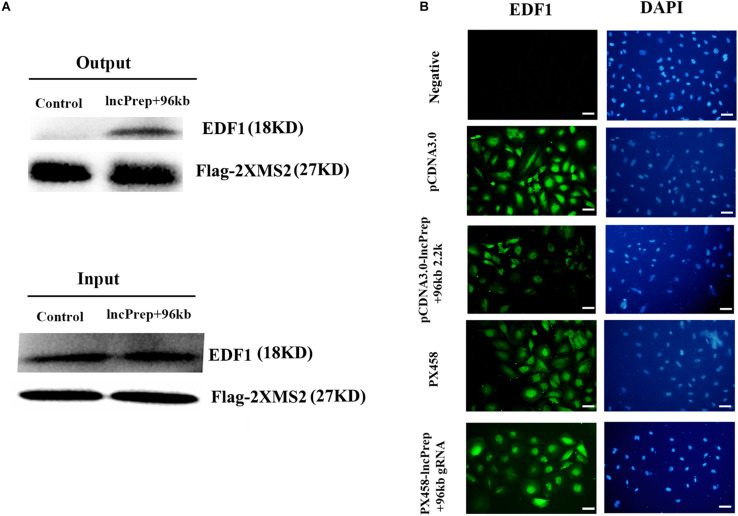
The effect of lncPrep + 96kb 2.2 kb on EDF1. **(A)** Western blot was used to determine the specific interaction of lncPrep + 96kb 2.2 kb with EDF1. **(B)** Immunofluorescence was used to examine the location of EDF1 in granulosa cells. Scale bar = 100 μm.

To identify lncPrep + 96kb 2.2 kb interacting proteins, we conducted RIP based on the specific interaction between the MS2 coat protein and the MS2 binding sites (MS2bs) and mass spectrometry analysis. Endothelial differentiation-related factor 1 was identified as a binding partner of lncprep + 96kb 2.2 kb. Western blotting with an anti-EDF1 antibody confirmed the existence of EDF1 within lncPrep + 96kb 2.2 kb pull-down samples (Figure 3A). We also observed the effect of lncPrep + 96kb 2.2 kb on EDF1 and found that overexpression of lncPrep + 96kb 2.2 kb induced the translocation of EDF1 from the nucleus to the cytoplasm, however, knockdown of lncPrep + 96kb 2.2 kb had no influence on the location of EDF1 (Figure 3B).

### EDF1-Specific shRNA Downregulates the Expression of Aromatase and Inhibits the Secretion of Estradiol

**FIGURE 4:**
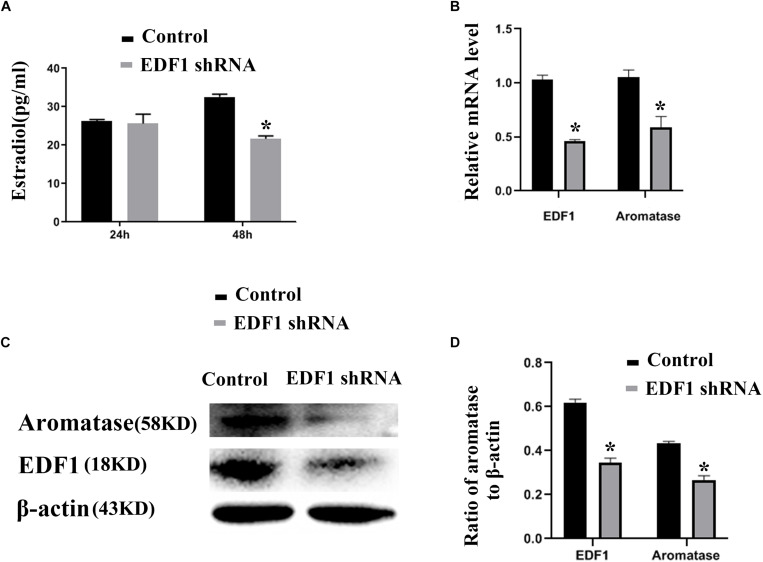
Estradiol production and aromatase expression are regulated by EDF1. **(A)** Shows the effect of EDF1-specific shRNA on estradiol production. **(B–D)** Shows the effect of EDF1-specific shRNA on aromatase expression at the mRNA and protein levels. The experiment was independently repeated for three times. *p < 0.05, which was a specific two-group comparison.

To confirm the function of EDF1 in murine ovaries, we knocked down the expression of EDF1 by EDF1-specific shRNA. After knockdown of EDF1, estradiol production and aromatase expression were decreased, suggesting that EDF1 promoted the secretion of estradiol (Figure 4).

### The 1,979–2,077 Site of lncPrep + 96kb 2.2 kb Contributes to the Binding of EDF1

**FIGURE 5:**
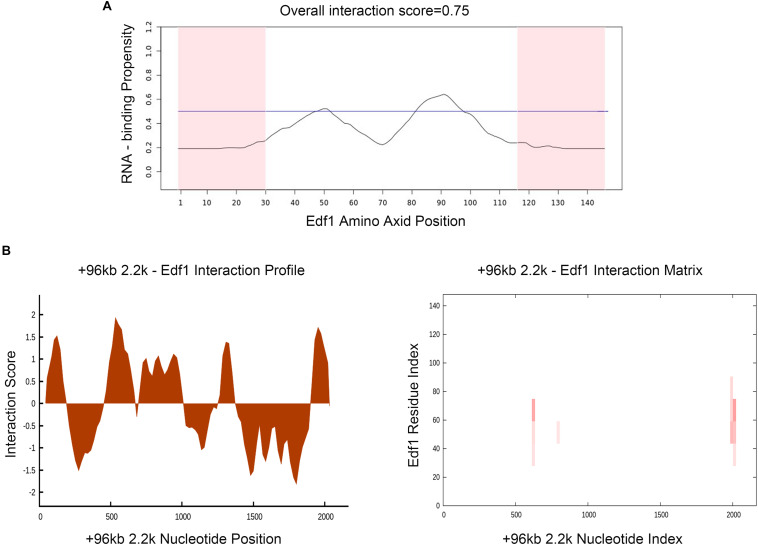
Prediction of the interaction between lncPrep + 96kb 2.2 kb and EDF1. **(A)** The RNA-binding capability of the EDF1 protein was predicted by the CatRAPID signature module. Overall interaction scores greater than 50% indicate binding capability. **(B)** CatRAPID fragment module prediction of the interaction profile and matrix for lncPrep + 96kb 2.2 kb and EDF1.

**FIGURE 6:**
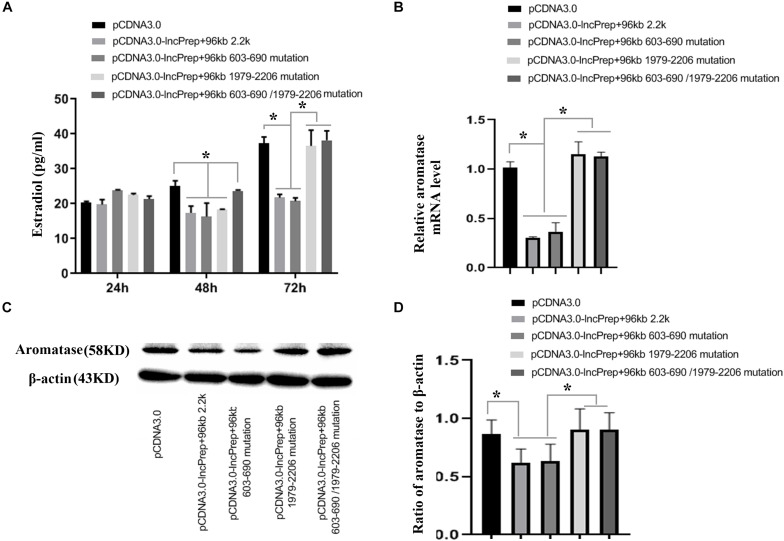
The effects of the mutating the lncPrep + 96kb 2.2 kb binding sites on estradiol production and aromatase expression. **(A)** Shows estradiol production after mutating the lncPrep + 96kb 2.2 kb binding sites. **(B)** Shows the expression of aromatase after mutating the lncPrep + 96kb 2.2 kb binding sites. **(C,D)** Show the protein expression of aromatase after mutating the lncPrep + 96kb 2.2 kb binding sites. *p < 0.05. The experiment was independently repeated for three times.

To prove the effect of lncPrep + 96kb 2.2 kb on aromatase and estradiol through EDF1, we first evaluated the possible interaction between lncPrep + 96kb 2.2 kb and EDF1 through bioinformatics approaches. The results from the determination of the CatRAPID signature revealed that the overall EDF1/RNA interaction score was 0.75 (Figure 5A). Then, the CatRAPID fragment algorithm, which is based on the interactions between individual polypeptides and nucleotide fragments, was used for analysis. As shown in Figure 5B, the 1,979–2,077 and 603–690 nucleotide positions of lncPrep + 96kb 2.2 kb have high capabilities to bind to EDF1. Then, we deleted these binding sites and found that mutation of 1,979–2,077 in lncPrep + 96kb 2.2 kb rescued the effects of lncPrep + 96kb 2.2 kb on estradiol production and aromatase expression. However, the 603–690 site did not contribute to the effect of lncPrep + 96kb 2.2 kb (Figures 6A–D).

## Discussion

Spatially and temporally specific expression of lncRNAs has a strong association with the cell differentiation process (Cesana et al., 2011; Wang Y. et al., 2013). LncPrep + 96kb is a novel lncRNA that is highly expressed in murine granulosa cells. We examined the spatially and temporally specific expression of lncPrep + 96kb through *in situ* hybridization. The results showed that lncPrep + 96kb was highly and specifically expressed in the granulosa cells of primary follicles and secondary follicles, and a low expression level was identified in antral follicles.

Early growth in follicular development is accompanied by an increase in oocyte diameter and the proliferation of granulosa cells. Early follicular development requires the appropriate expression of numerous genes at different developmental stages (Bonnet et al., 2013, 2015; Ernst et al., 2018). LncPrep + 96kb was specifically expressed in granulosa cells of early stage follicles, so we inferred that it may influence the proliferation of granulosa cells. However, the results did not support this hypothesis. Compared with the granulosa cells of early growing follicles, the granulosa cells of antral follicles express different differentiation markers, such as FSH receptor and aromatase, and have the capacity to secrete estradiol. We cultured granulosa cells and observed the effect of lncPrep + 96kb 2.2 kb on estradiol production. We found that lncPrep + 96kb 2.2 kb inhibited estradiol production and aromatase expression. These results suggest that high expression of lncPrep + 96kb 2.2 kb may maintain the granulosa cells of early stage follicles in an undifferentiated state.

As the key enzyme of estradiol synthesis, aromatase controls the production of estradiol. In adult rats, aromatase is no longer expressed by small growing follicles, but is confined to healthy large antral follicles and preovulatory follicles (Guigon et al., 2003; Sakurada et al., 2006; Stocco, 2008; Dewailly et al., 2016). The spatially and temporally specific expression of aromatase was strictly controlled. The proximal ends of aromatase promoters contain a number of fairly well-conserved regulatory elements. The ovary-specific PII promoter binds to cAMP-response element-binding protein (CREB), LRH1/NR5A2, and SF1/NR5A1 (Stocco, 2008; Lai et al., 2014). LRH1 is primarily expressed in granulosa cells and luteal cells, whereas SF1 is expressed in theca/interstitial cells and granulosa cells (Falender et al., 2003; Hinshelwood et al., 2003; Liu et al., 2003; Lai et al., 2014). Therefore, in granulosa cells, SF1 and LRH1 contribute to the regulation of aromatase.

As unstable biomacromolecules, lncRNAs bind to specific proteins to form RNA/protein complexes to ensure their stability in cells. In addition, the biological function of lncRNAs cannot be achieved without the reversible or irreversible binding of related protein molecules. RNA–protein interactions are the basis of many cellular biological processes, such as transcription, posttranscriptional regulation, protein translation, RNA stability, and RNA transport and localization. Long non-coding RNAs can interact with proteins in a variety of forms to serve as protein decoys and protein scaffolds to perform functions related to protein targeting and intracellular signaling (Wang and Chang, 2011). We employed RIP based on the specific interaction between the MS2 coat protein and MS2 binding sites (MS2bs) to identify the proteins that bind to lncPrep + 96kb 2.2 kb. Mass spectrometry and Western blotting identified EDF1, which can bind to lncPrep + 96kb 2.2 kb. Further study showed that overexpression of lncPrep + 96kb 2.2 kb induced the translocation of EDF1 from the nucleus to the cytoplasm.

Endothelial differentiation-related factor 1, a highly conserved intracellular protein, was initially identified as an evolutionarily conserved transcriptional coactivator that links regulatory factors and the TATA element-binding protein (Takemaru et al., 1997). Studies have shown that EDF1 can act as a bridging factor, enabling the interaction of SF1, LRH1, liver-X-receptor-α (LXR-α), and peroxisome proliferator activated receptor-γ with the transcription machinery (Brendel et al., 2002; Leidi et al., 2009). As a transcriptional coactivator, EDF1 functions in the nucleus of cells and interacts with TATA box-binding proteins. Some studies also showed that EDF1 was localized in the cytosol, where it binds to calmodulin and regulates CaM availability (Ballabio et al., 2004; Cazzaniga et al., 2018). Therefore, EDF1 serves different functions depending on the location of EDF1 in cells. Recent studies have shown that the lncRNA Blnc1 can bind to EDF1 and contribute to LXR transcriptional activation. According to the RIP results, we also showed that EDF1 can bind to lncPrep + 96kb 2.2 kb, and lncPrep + 96kb 2.2 kb promoted the translocation of EDF1 from the nucleus to the cytoplasm. We tested the function of EDF1 in granulosa cells and found that EDF1-specific shRNA indeed inhibited estradiol production and aromatase expression. Endothelial differentiation-related factor 1 is a coactivator of SF1 and LRH1, which are the main regulators of the aromatase promoter. Therefore, we infer that EDF1 contributes to estradiol production in granulosa cells as a coactivator. Before prepuberty, lncPrep + 96kb 2.2 kb binds to EDF1 and remains in the cytoplasm to prevent the activity of EDF1 as a coactivator.

Subsequently, the binding sites of lncPrep + 96kb 2.2 kb and EDF1 were predicted by CatRAPID. Two locations in lncPrep + 96kb 2.2 kb (1,979–2,077 and 603–690) had high scores. After mutation of these two locations, we found that the loss of 1,979–2,077 but not 603–690 reduced the effect of lncPrep + 96kb 2.2 kb on estradiol production and aromatase expression. These results suggested that the binding of lncPrep + 96kb 2.2 kb with EDF1 contributed to the localization of EDF1 and inhibited aromatase expression. We will explore the function of EDF1 in the cytoplasm in future studies.

## Conclusion

Taken together, the results of this study demonstrate the function of lncPrep + 96kb 2.2 kb in granulosa cells. This lncRNA was highly expressed in granulosa cells of early growing follicles. It inhibited aromatase expression and estradiol production in granulosa cells by inducing the translocation of EDF1 from the nucleus to the cytoplasm.

## Data Availability Statement

All datasets generated for this study are included in the article/Supplementary Material.

## Ethics Statement

The animal study was reviewed and approved by the Ethical Committee of Nanchang University.

## Author Contributions

FF, JW, and RB performed the experiments and wrote the manuscript. LL, XT, SH, HZ, WW, and LX performed the experiments and analyzed the data. CZ conceived and designed the experiments. All authors contributed to the article and approved the submitted version.

## Conflict of Interest

The authors declare that the research was conducted in the absence of any commercial or financial relationships that could be construed as a potential conflict of interest.

## Footnotes

1 http://crispr.mit.edu

## Supplementary Material

FIGURE S1 The purity of granulosa cells. The expression of FSHR was detected with immunofluorescence to confirm the purity of granulosa cells. We examined the FSHR signal in granulosa cells isolated from PMSG treated mice through mechanical isolation. Scale bar = 100 μm.

FIGURE S2 The effect of lncPrep + 96kb 2.2 kb on StAR, P450scc, SF and GATA4. Western blotting was used to examine the protein level of StAR, P450scc, SF and GATA4 after overexpression and knockdown of lncPrep + 96kb 2.2 kb.

FIGURE S3 The effect of lncPrep + 96kb 2.2 kb on estradiol production and aromatase expression in granulosa cells from untreated mice. The granulosa cells were isolated from 21 days female mice that were not treated with PMSG. After the overexpression and knockdown of lncPrep + 96kb 2.2 kb, we examined the estradiol production at 72 h and aromatase expression at 48 h. **(A)** shows estradiol production after the overexpression and knockdown of lncPrep + 96kb 2.2 kb. **(B)** shows the mRNA expression of aromatase after overexpression and knockdown of lncPrep + 96kb 2.2 kb. **(C)** shows the protein expression of aromatase after overexpression and knockdown of lncPrep + 96kb 2.2 kb. ^∗^*p* < 0.05.
