# Supplementary figures and images for: LncPrep + 96kb 2.2 kb Inhibits Estradiol Secretion From Granulosa Cells by Inducing EDF1 Translocation
Source: Front Cell Dev Biol. 2020 Jun 30;8:481. doi: 10.3389/fcell.2020.00481 (PMC7338311; doi:10.3389/fcell.2020.00481)

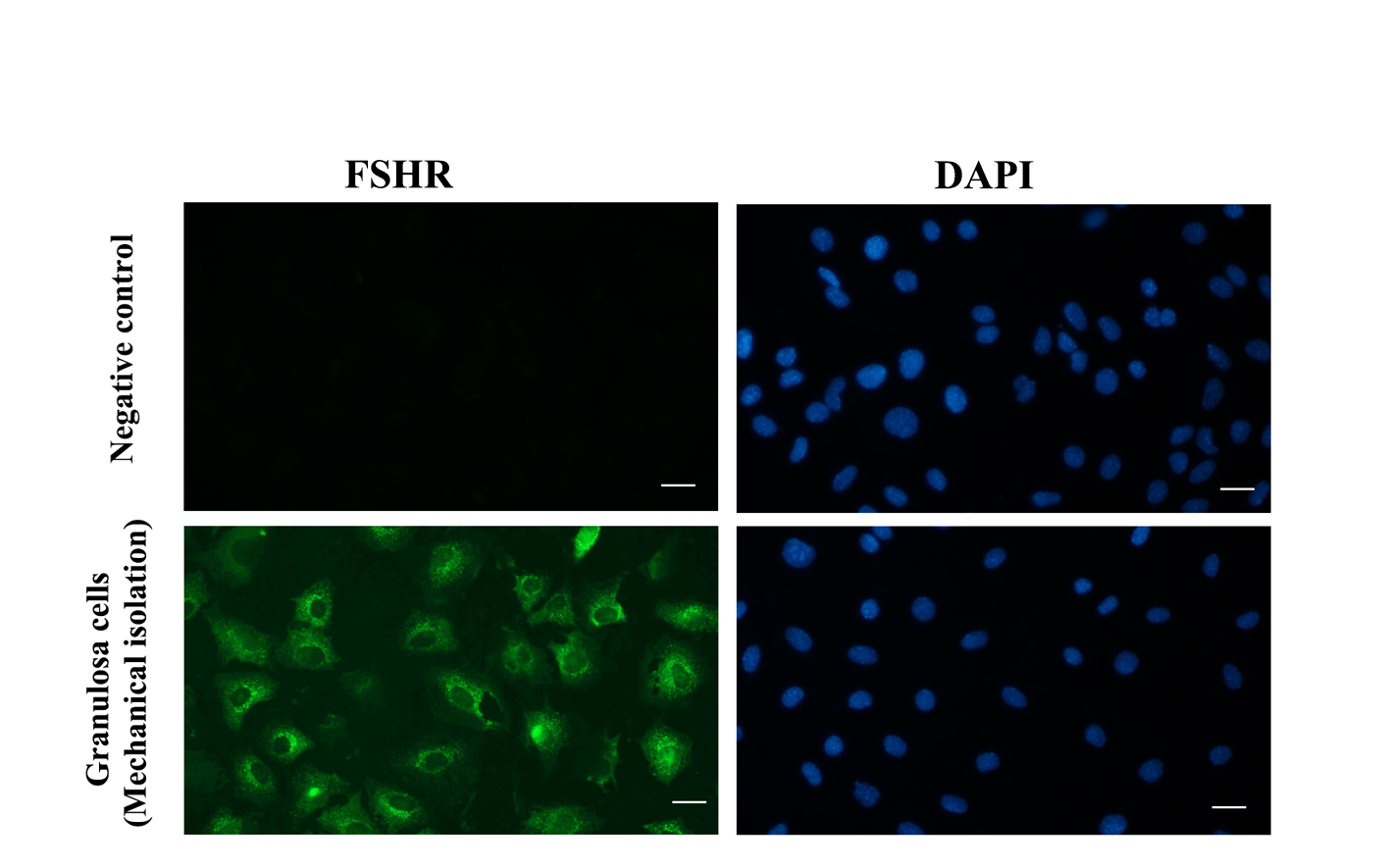

Supplement: FIGURE S1 — The purity of granulosa cells. The expression of FSHR was detected with immunofluorescence to confirm the purity of granulosa cells. We examined the FSHR signal in granulosa cells isolated from PMSG treated mice through mechanical isolation. Scale bar = 100 μm. [file Image_1.TIF]

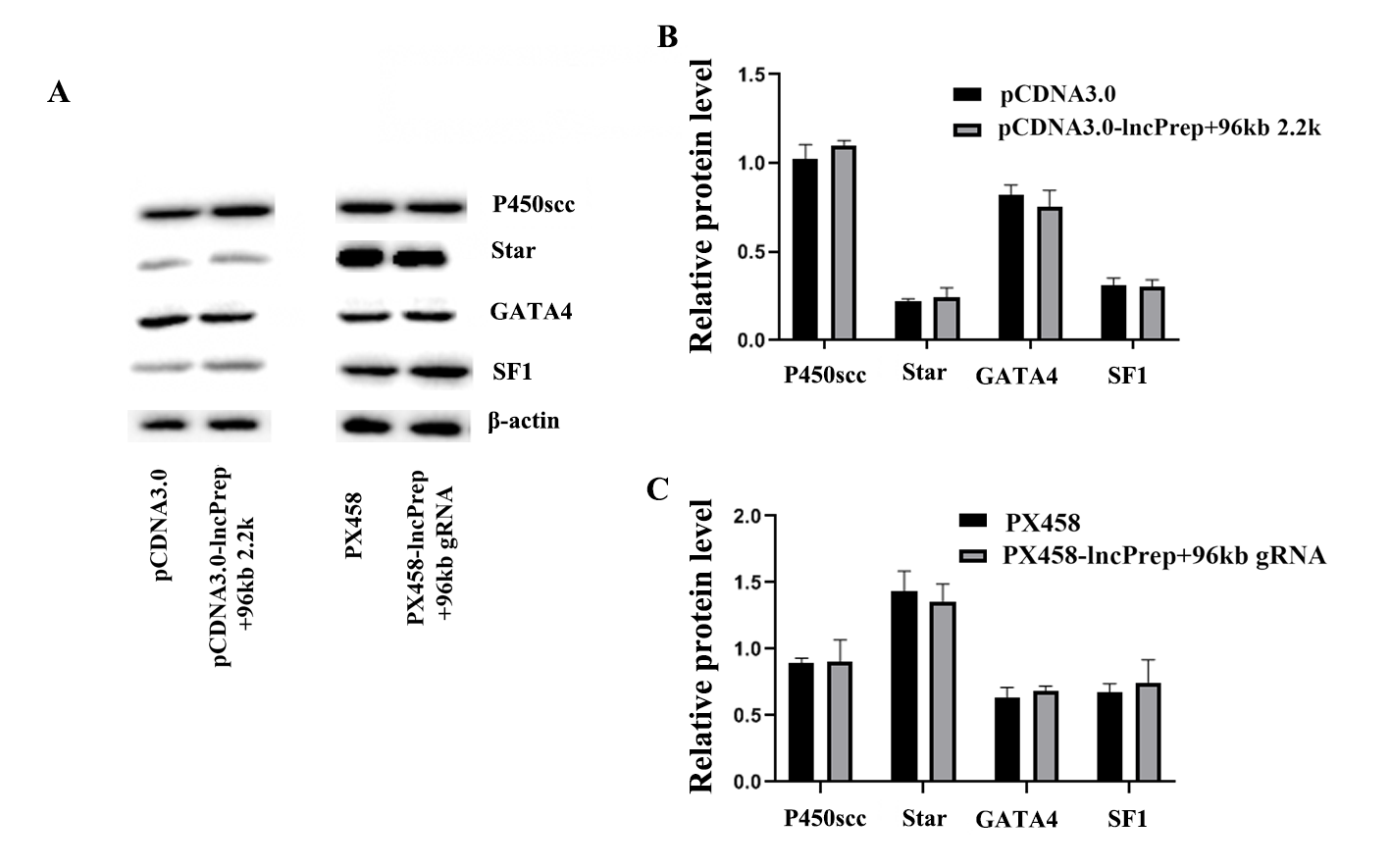

Supplement: FIGURE S2 — The effect of lncPrep + 96kb 2.2 kb on StAR, P450scc, SF and GATA4. Western blotting was used to examine the protein level of StAR, P450scc, SF and GATA4 after overexpression and knockdown of lncPrep + 96kb 2.2 kb. [file Image_2.TIF]

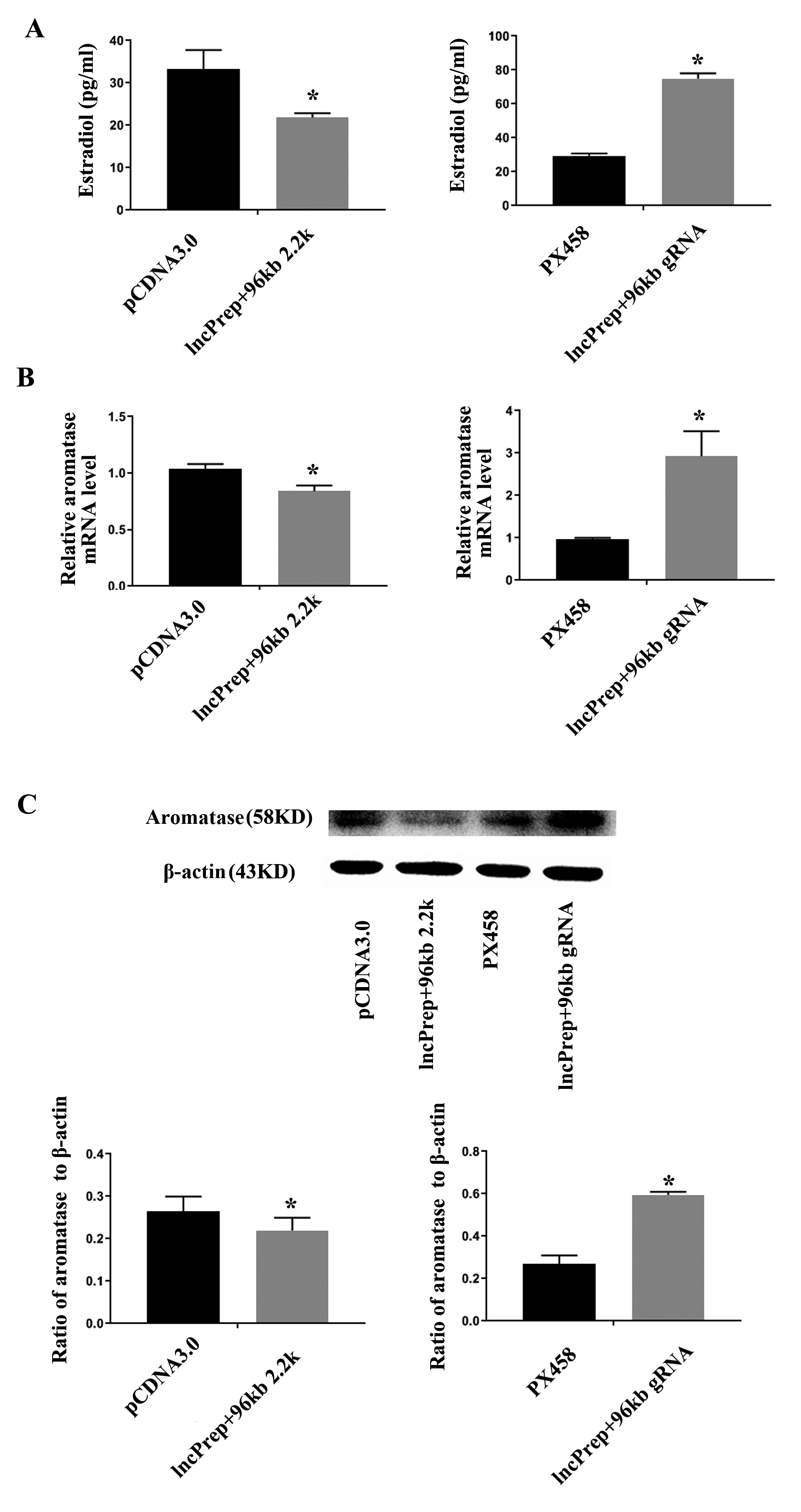

Supplement: FIGURE S3 — The effect of lncPrep + 96kb 2.2 kb on estradiol production and aromatase expression in granulosa cells from untreated mice. The granulosa cells were isolated from 21 days female mice that were not treated with PMSG. After the overexpression and knockdown of lncPrep + 96kb 2.2 kb, we examined the estradiol production at 72 h and aromatase expression at 48 h. (A) shows estradiol production after the overexpression and knockdown of lncPrep + 96kb 2.2 kb. (B) shows the mRNA expression of aromatase after overexpression and knockdown of lncPrep + 96kb 2.2 kb. (C) shows the protein expression of aromatase after overexpression and knockdown of lncPrep + 96kb 2.2 kb. ∗p < 0.05. [file Image_3.TIF]
